# Two-Step Amyloid Aggregation: Sequential Lag Phase Intermediates

**DOI:** 10.1038/srep40065

**Published:** 2017-01-09

**Authors:** Fabio Castello, Jose M. Paredes, Maria J. Ruedas-Rama, Miguel Martin, Mar Roldan, Salvador Casares, Angel Orte

**Affiliations:** 1 Dept. of Physical Chemistry, Faculty of Pharmacy, University of Granada, Cartuja Campus, 18071, Granada Spain; 2 GENYO, Pfizer-University of Granada-Junta de Andalucia Centre for Genomics and Oncological Research, Avda Ilustracion 114. PTS, 18016, Granada Spain; 3 Dept. of Physical Chemistry, Faculty of Sciences, University of Granada, Fuentenueva Campus, 18071, Granada Spain

## Abstract

The self-assembly of proteins into fibrillar structures called amyloid fibrils underlies the onset and symptoms of neurodegenerative diseases, such as Alzheimer’s and Parkinson’s. However, the molecular basis and mechanism of amyloid aggregation are not completely understood. For many amyloidogenic proteins, certain oligomeric intermediates that form in the early aggregation phase appear to be the principal cause of cellular toxicity. Recent computational studies have suggested the importance of nonspecific interactions for the initiation of the oligomerization process prior to the structural conversion steps and template seeding, particularly at low protein concentrations. Here, using advanced single-molecule fluorescence spectroscopy and imaging of a model SH3 domain, we obtained direct evidence that nonspecific aggregates are required in a two-step nucleation mechanism of amyloid aggregation. We identified three different oligomeric types according to their sizes and compactness and performed a full mechanistic study that revealed a mandatory rate-limiting conformational conversion step. We also identified the most cytotoxic species, which may be possible targets for inhibiting and preventing amyloid aggregation.

Protein misfolding is the result of an alteration of the normal protein folding process. Incorrectly folded proteins frequently form aggregates with different morphologies, from amorphous structures to highly organized fibrillar aggregates called amyloid plaques. The presence of insoluble amyloid deposits in damaged tissues is a feature of approximately forty neurodegenerative and systemic pathologies of great socioeconomic importance, including Alzheimer’s, Parkinson’s and Huntington’s diseases and diabetes mellitus type 2^1^. These deposits are composed of proteins that exhibit similar characteristics, such as a fibrillar morphology, a prevalence of β-sheet structures, and a diameter of approximately 10 nm^1^. Despite the similarity of the final forms, the fibrils originate from peptides or protein groups that are not structurally or functionally related. Despite active research in the field, the mechanism of amyloid fibril formation remains controversial because parallel pathways may lead to aggregation, including an orderly association of oligomers or a multi-step process in which certain prenucleation clusters act as an elongation fulcrum for the formation of the fibrillar forms^2^,^3^,^4^. The importance of understanding the prefibrillar forms involved in these early phases is emphasized by their crucial role in neurotoxicity^5^,^6^. Hence, the study of the aggregation process is currently focused on determining the roles of these intermediates as well as their complete characterization^3^,^7^ because they are potential targets that could define molecular treatment strategies.

These early stages of aggregation represent a challenge for conventional biophysical techniques because these techniques only provide average information about the ensemble of the population and cannot provide insight into the heterogeneity of the samples. Similarly, conventional biophysical techniques exhibit relatively low sensitivity, setting specific concentration requirements for the measurements. This requirement frequently leads to studies of protein aggregation using high protein concentrations that are usually far from physiologically relevant ranges. For instance, amyloid-β is present at nM levels under physiological conditions^8^,^9^. However, local increases in the concentration to μM levels by membrane association^10^, macromolecular crowding^11^, or accumulation in cell organelles^12^ have been suggested as potential mechanisms to initiate the aggregation cascade of amyloid-β. However, these *supra*-physiological concentrations in the μM regime remain too low for some of conventional biophysical techniques. Therefore, studying the processes underlying the initial stages of amyloid aggregation requires techniques with molecular resolution. In particular, innovative computational simulations and single-molecule fluorescence (SMF) are enriching our knowledge of amyloid assembly from a molecular perspective. For example, *in silico* experiments have suggested the importance of the hydrophobically driven coalescence of monomers prior to conformational conversion into aggregates that are capable of causing amyloid proliferation^2^,^13^. In SMF experiments, excitation lasers are focused on a small diffraction-limited spot using the powerful optics of a confocal microscope. Individual fluorescently labelled proteins can then be detected when they cross into and out of the excitation probe volume via Brownian diffusion. The compatibility of SMF techniques with low concentrations represents an important advantage that permits closer observation of the aggregation process. Advanced dual-colour SMF experiments have provided insights into the aggregation of disease-related proteins, such as neuroserpins^14^, α-synuclein^5^,^15^,^16^, or amyloid-β^17^,^18^. For instance, Cremades and colleagues showed that α-synuclein populates two different types of oligomers in the on-pathway of aggregation^5^ and that different disease-related single-point mutations may alter the relative populations of these types and cause the acceleration or slowing of the fibril formation process^15^.

One of the major strategies in protein aggregation research is the use of protein model systems that are not strictly associated with any particular disease but demonstrate aggregation that evolves into amyloid formation, thus representing a valuable tool for drawing generalized conclusions. Among the suitable protein models, SH3 domains have been extensively used in amyloid studies because of their ability to form amyloid aggregates under certain pH, temperature and ionic strength conditions^19^. In particular, we have focused our attention on the N47A variant of the *α*-spectrin SH3 domain (N47A-SH3), which exhibits enhanced aggregation behaviour compared with the wild type domain^20^ and has been broadly explored under bulk, high-concentration conditions^21^. In a previous work, we employed a dual-laser, multi-dimensional SMF technique to investigate the initial step of amyloid oligomerization using a labelled N47A-SH3 domain^22^. At the single-molecule level, we found evidence of very rapid but nonspecific oligomerization in the absence of incubation. Dissolving the N47A-SH3 protein in an amyloid-promoting buffer only triggered the formation of aggregates in dynamic equilibrium with the monomeric forms. Our single-molecule experiments allowed us to quantify the thermodynamic constant for this first equilibrium.

In the present work, we investigated the mechanism of amyloid oligomerization by the N47A-SH3 domain at the molecular level by further evaluating the progress of these aggregates. Our work, which is well supported by other bulk biophysical techniques, provides insights on the intimate mechanism of oligomerization toward the formation of amyloid fibrils. Specifically, we provide experimental evidence for a two-step nucleation mechanism for the formation of aggregates that evolve into mature fibrils at low *supra*-physiological concentrations.

## Results

### SMF characterization of amyloidogenic oligomers

Previous studies of the N47A-SH3 aggregate cascade were performed at high (>0.36 mM) protein concentrations to overcome the lag phase, although only a single nucleation step was detected kinetically. However, we sought to investigate the low concentration regime, which emulates the slow aggregation conditions observed in amyloid diseases. Under these conditions, *in silico* studies have reported the crucial role of nonspecific contacts as the initial step in aggregation^2^. We labelled the N47A-SH3 domain with an optimal dye pair^22^ that is capable of undergoing resonance energy transfer (FRET) when in close proximity to study these processes and the corresponding amyloidogenic oligomers using SMF techniques. We employed either ATTO 488 (A488 as the donor) or ATTO 647N (A647N as the acceptor) to label the N47A-SH3 monomers on a designed N-terminal cysteine residue located after a flexible six-residue (Gly-Ser-Gly-Ser-Gly-Cys) tail. The Förster distance, *R*_0_, for this pair of fluorophores is 51 Å^23^, which allowed us to detect the formation of aggregates at the single-molecule level based on the FRET efficiency, *E*, when the labelled monomers interacted with each other^22^. The incubation of equimolar mixtures of the donor- and acceptor-labelled monomers (N47A-SH3-DA) under amyloidogenic conditions (0.1 M glycine and 0.1 M NaCl, pH 3.2, 37 °C, hereafter referred to as ‘aggregation buffer’) for several hours resulted in the effective co-aggregation and formation of mature fibrils (see Supplementary Information, SI, and Supplementary Fig. S1). The presence of the tail and the fluorophores did not inhibit the ability of the protein to form amyloid fibrils and did not alter the initial state (native structure) or the final state (curly amyloid fibrils) of the aggregation process of the N47A-SH3 domain^22^. Compared with the unlabelled control, the shape of the fibrils was curly in both cases, which is typical for heterogeneous nucleation^21^, although the aggregation of the labelled protein was faster than expected because of the enhanced aromaticity conferred by the pair of fluorophores^24^.

**Figure 1:**
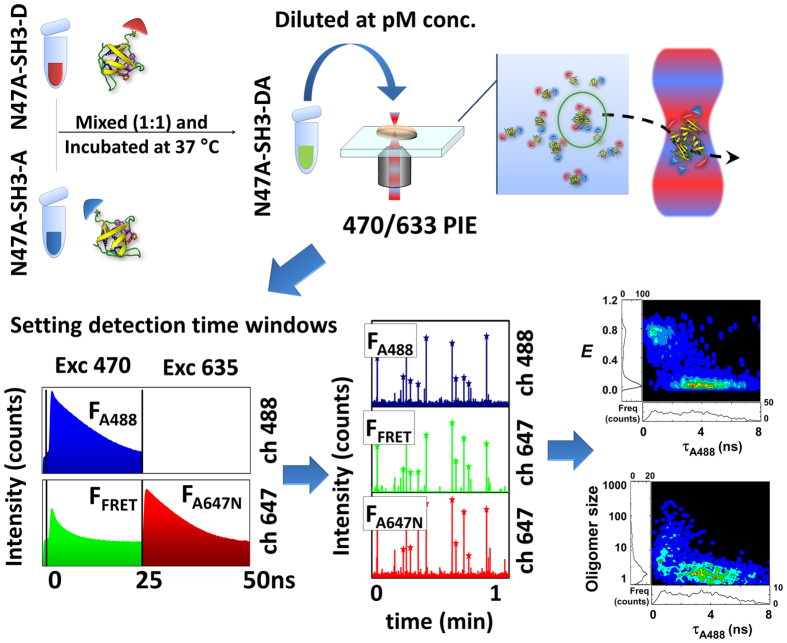
SMF-PIE scheme. The interleaved excitation allowed us to allocate specific time windows for each excitation source, reconstructing the fluorescence decay to extract the lifetimes. Fluorescence was split into two channels to detect the fluorescence from the A488 (ch 488) and the A647N fluorophores (ch 647). Three simultaneous traces were defined: donor, FRET, and directly excited acceptor. Several correlograms of the different parameters were constructed to extract the maximum information of the individual molecules.

We studied the early stages of amyloid oligomerization of the N47A-SH3-DA samples in aliquots at different incubation times by analyzing the samples using SMF pulsed interleaved excitation (SMF-PIE, see SI and Fig. 1). Oligomers were sensitively detected in the fluorescence traces as molecules containing at least one A488- and one A647N-labelled monomer, even in the presence of a large excess of monomers^22^,^25^. SMF-PIE is a powerful, dual-colour, multi-dimensional method that is capable of extracting a variety of parameters, such as the fluorescence lifetime of individual molecules, τ, or burst-wise FRET efficiency, *E*, with much higher reliability than conventional single-molecule FRET experiments^25^,^26^. These parameters can be correlated for each individual molecule event, thus allowing the construction of multi-dimensional population histograms (Fig. 1).

For each individual aggregate detected, we estimated the (burst-wise-obtained) FRET efficiency, *E*, the fluorescence lifetime of the donor, τ_A488_, and the apparent oligomer size. τ_A488_ entails an orthogonal estimation of the FRET efficiency through the quenching caused by the A647N fluorophore because *E* = 1 − τ_A488_/τ_0_, where τ_0_ is the fluorescence lifetime of the donor in the absence of the acceptor, which is 4.0 ns for A488^23^. A decrease in τ_A488_ indicates efficient FRET because the parameter τ_A488_ is advantageously free of issues such as crosstalk and different detection efficiencies between channels. For the apparent oligomer size, the total fluorescence intensity as the sum of fluorescence intensity of the directly excited donor and the acceptor was converted to the number of monomer molecules by normalization to the average fluorescence intensity of a monomer^25^; however, access to the value of τ_A488_ permitted us to implement a correction on the oligomer size caused by the direct quenching of the A488 fluorescence (see Methods and SI). This important correction has been introduced here for the first time, in contrast to the systematic potential underestimation of the oligomer size in previous studies^17^,^25^.

**Figure 2:**
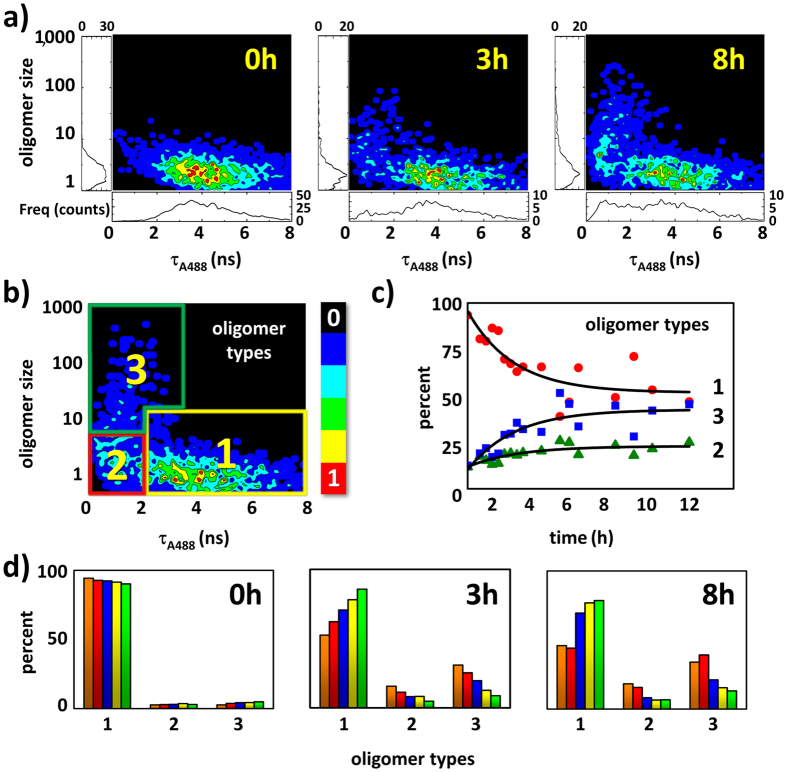
Single-molecule experiments on incubated N47A-SH3-DA. (**a**) Oligomer size vs. τ_A488_ single-molecule correlograms from 32 μM samples after 0, 3, and 8 h of incubation. (**b**) Definitions of the three types of oligomers: **1**, **2**, and **3**. (**c**) Time trace of the relative populations of each type of oligomer (**1**, **2**, and **3**) from the incubated 32 μM samples. (**d**) Relative populations of the oligomers after 0, 3, and 8 h of incubation of the samples at total protein concentrations of 44 (orange), 32 (red), 28 (blue), 24 (yellow), and 20 μM (green).

The *E* vs. τ_A488_ correlograms revealed the presence of a population of soluble oligomers at early times as reported previously^22^ and a distinct high FRET population arising with the incubation time (Supplementary Fig. S2) that displayed higher values of FRET efficiency. However, more interesting and stimulating results emerged from the correlation of the oligomer size with the FRET efficiency based on τ_A488_. Figure 2a and Supplementary Fig. S3 show the oligomer size vs. τ_A488_ correlograms at different incubation times and different total protein concentrations. These analyses uniquely allowed us to directly identify three types of oligomers and follow their kinetic history upon aggregation. At the very early moments of aggregation, type **1** oligomers exhibited a low FRET efficiency (with τ_A488_ ≈ 3–4 ns, *E* = 0.00–0.25) and small size (4–10 monomer units). In a previous work, we estimated the apparent dissociation equilibrium constant for these type **1** oligomers as 10.1 ± 0.9 nM^22^, indicating their high stability under these experimental conditions. Prior to the formation of large aggregates, we detected the development of a second compact oligomer population (type **2**) whose size was comparable to the initial population (4–10 monomeric units) but that displayed a high FRET efficiency as evidenced by a reduced τ_A488_ value efficiency (τ_A488_ ≈ 1–2 ns, *E* = 0.50–0.75), thus supporting a more compact structure of the aggregates. At longer incubation times, a third compact population of large oligomers (from 20 to over 100 monomers) that also exhibits high FRET efficiency clearly arose (type **3**). The distinction between types **2** and **3** was not detectable in the *E* vs. τ_A488_ correlograms because the heterogeneity between populations was only accessible from the different total fluorescence intensities of the events. Importantly, even for fibrillar samples incubated for several days, the supernatant always contained a mixture of oligomers of types **1**, **2** and **3**. This observation suggests a continuous dynamic equilibrium governing the three states and supports the potential function of mature fibrils as a source of readily formed oligomers^17^.

We quantified the relative contribution of each type of oligomer by defining appropriate threshold regions in the correlograms (Fig. 2b). Using the number of events under each region, we estimated the relative amounts of oligomer types **1**, **2**, and **3** and followed their variation with incubation time (Fig. 2c and d). At the highest concentrations studied (32 and 44 μM), the appearance of types **2** and **3** was evident even after 1 h of incubation. Samples incubated at lower concentrations (20 and 24 μM) exhibited a slower growth of the compact oligomer forms. With this quantitative information, we performed a thorough kinetic and mechanistic study to understand the roles of these species in amyloid fibril formation.

### Kinetic analysis of the two-step amyloid nucleation

**Figure 3:**
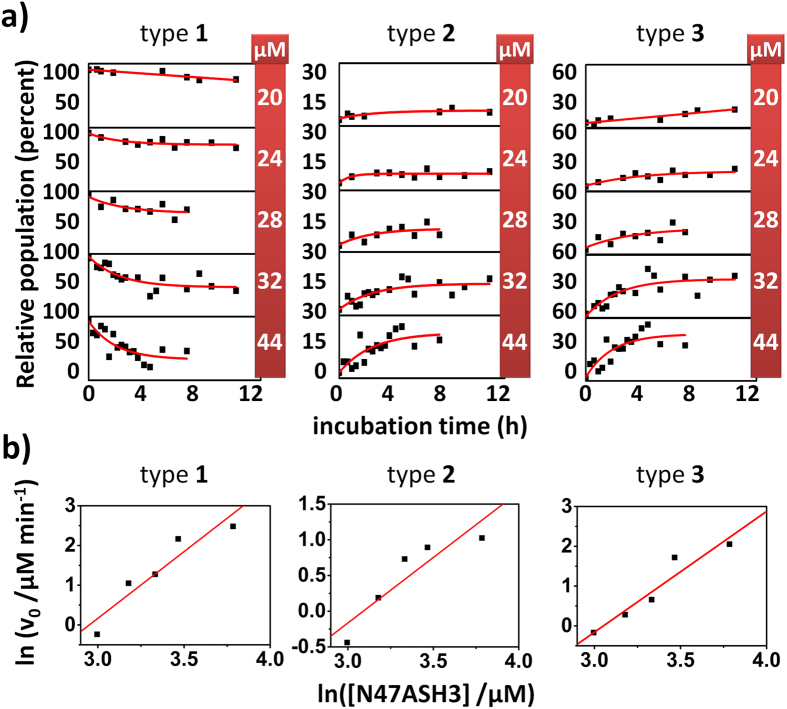
Kinetic study of each type of oligomer using the initial rates method. (**a**) Time evolution of the relative populations of types **1**, **2**, and **3** oligomers at total N47A-SH3-DA concentrations of 20, 24, 28, 32 and 44 μM. The red lines represent the fits using single exponential functions. The data points are derived from three independent incubations. (**b**) Initial rates, *v*_0_, of the disappearance (type **1**) and formation (types **2** and **3**) of oligomers as a function of the total protein concentration, on the logarithmic scale. The red lines represent linear fits.

We performed a thorough kinetic analysis of the disappearance and formation of the different types of oligomers at the single-molecule level using the method for determining the initial rates at different total protein concentrations (see SI). From the exponential fits of the relative populations of the oligomers (Fig. 3a), the initial rates of the disappearance (type **1**) and formation (types **2** and **3**) of the oligomers were obtained (Supplementary Table S1). The apparent order of the reaction was obtained from the slope of the logarithmic plot of the initial rates vs. the total protein concentration (ln(v_0_) vs. ln[N47A-SH3], Fig. 3b). The apparent value of the order of reaction was (2.9 ± 0.7 SEM) for the disappearance of the type **1** oligomers but (1.8 ± 0.4 SEM) and (3.2 ± 0.7 SEM) for the formation of types **2** and **3**, respectively. The limiting step among these processes is the formation of the type **2** oligomers, which exhibited the slowest rates at any concentration tested (Supplementary Table S1). The apparent reaction order of the formation of type **2** oligomers was close to 2 with respect to the total protein concentration. To understand this result, we assumed a first-order conformational rearrangement type **1** → type **2**. This reaction may be interpreted as the formation of β-sheet structures. The initial rate of formation of type **2** is given by *k*_1→2_·[1]_0_, where [1]_0_ is the concentration of type **1** oligomers at the beginning of the aggregation reaction. In contrast to other previous kinetic studies of amyloid formation at the single-molecule level^27^, for this N47A-SH3 domain, there is always certain concentration of type **1** oligomers in equilibrium, and hence [1]_0_ ≠ 0. The initial concentration of type **1** oligomers is determined by the equilibrium, which we demonstrated to follow an apparent equilibrium constant with the form *K*_*a*_ = [1]/[M]^2^, where [M] is the monomer concentration^22^. Therefore, we can write that the initial rate of formation of type **2** oligomers is given by *k*_1→2_·*K*_*a*_·[M]_0_^2^, which unequivocally explains the apparent order 2 for the formation of type **2** oligomers with respect to the total protein concentration. This dependence indicates that the formation of type **2** oligomers is mainly determined by a first-order conformational change of the type **1** oligomers. Moreover, this reaction order is consistent with the results reported in a kinetic study by Ruzafa and colleagues^21^, who used far-UV CD at a protein concentration range of 0.36–1.11 mM. The disappearance of the type **1** oligomers exhibits important concentration-dependent kinetics that corresponds to an apparent third-order reaction, thus indicating that the conformational rearrangement of type **1** into type **2** oligomers is not the only pathway of disappearance. Therefore, species that formed at later stages may be involved in the cooperative incorporation of the type **1** oligomers into mature aggregates. The formation of type **3** oligomers also showed a remarkable concentration dependence and a similar reaction order as the disappearance of the type **1** oligomers. The type **3** population was the last to appear, although it exhibited a more rapid formation rate than the type **2** oligomers. This result indicates that the formation of large aggregates during the growth phase of the sigmoidal kinetic aggregation process is not the limiting step and proceeds easily once nucleation is finished. Considering that the most relevant process to form type **3** from type **2** oligomers, at the initial times, must be monomer addition as it represents the largest probability of encounters, an apparent third-order rate of formation of type **3** oligomers may be expected (since the initial rate of formation of type **2** follows an apparent second order with respect to [M]_0_).

Hence, aggregate growth requires two types of nuclei, both type **1** and type **2** aggregates. This two-step model for amyloidogenic nucleation implies experimental evidence of the mechanism suggested by Knowles and colleagues for aggregation at low concentrations, which was obtained through computer simulations^2^. Similarly, our results confirm the importance of nonspecific interactions (type **1** oligomers) for amyloid growth. The necessary conversion step from type **1** to type **2** oligomers that we observed for the N47A-SH3 oligomers and that led to the formation of larger oligomers is also consistent with the kinetic model for α-synuclein aggregation reported by Iljina and colleagues^27^. These authors used the *master equation* formalism^28^ to interpret the kinetics of amyloid formation of α-synuclein and applied a nucleation-conversion-polymerization model to fit their experimental data. Considering either unimolecular or bimolecular conversion models, they assume first- or second-reaction orders for the conversion from low FRET to compact high FRET oligomers, respectively. Although these authors reported that the first-order conversion provided slightly better results, they also claimed that the difference between the two models was not completely clear. In our study, in which no *a priori* model formalisms were used, the nucleation reaction orders obtained from our experimental data were comparable to the orders observed by Iljina and colleagues. In addition, we obtained evidence showing that the conversion from type **1** oligomers to the more compact type **2** oligomers mainly accounted for a first-order conformational rearrangement. Moreover, Iljina and colleagues suggested first-order kinetics for the final conversion from high FRET-efficiency oligomers to fibrils, whereas our results suggest higher-order kinetics for the conversion from type **2** oligomers to larger type **3** oligomers.

**Figure 4:**
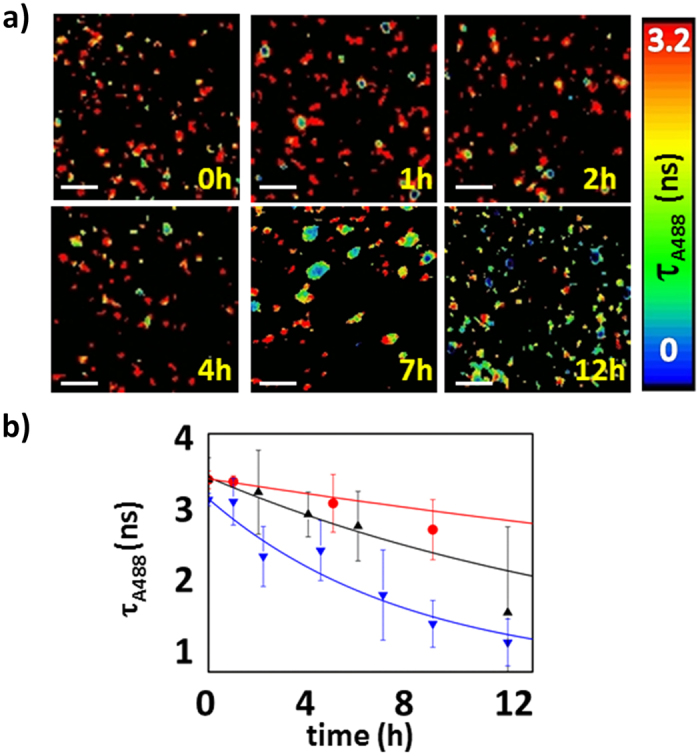
FLIM studies of the aggregation of N47A-SH3-DA. (**a**) A488 FLIM images of N47A-SH3-DA incubated at 32 μM. Aliquots were collected at different incubation times and diluted to 280 pM for imaging. The white scale bars represent 2.4 μm. (**b**) Average τ_A488_ values vs. incubation time from the FLIM images of aliquots of the N47A-SH3-DA mixture at 24 (red), 28 (black), and 32 μM (blue). The error bars represent the s.d. of the τ_A488_ values obtained from at least 10 different FLIM images. The lines indicate the fits to a single-exponential function.

We employed an advanced fluorescence lifetime imaging microscopy with pulsed interleaved excitation scheme (FLIM-PIE, see SI) to obtain supporting evidence for our conclusions regarding the formation of the three types of oligomers, their time evolutions, and their dependence on the protein concentration. FLIM-PIE is an imaging technique with single-molecule resolution that allows precise identification of oligomers (through the simultaneous reconstruction of the donor, FRET, and directly excited acceptor images, Supplementary Fig. S4) and the estimation of intra-oligomer FRET efficiency (through the donor fluorescence lifetime, τ_A488_). Using this technique, we studied the dynamics of N47A-SH3-DA aggregation at different incubation times and concentrations. The A488 FLIM images clearly showed two important features: the presence of small, high-FRET aggregates and growth into large aggregates. Figure 4a and Supplementary Fig. S5 show examples of A488 FLIM images at different total protein concentrations and incubation times. We also explored the time evolution of the A488 average lifetime, τ_A488_, within the detected oligomers at different N47A-SH3-DA concentrations (Fig. 4b). We observed a concentration-dependent decrease in the τ_A488_ values with incubation time. The decrease in τ_A488_ was fitted to single-exponential decay curves to extract the apparent decay times and rate constants at each total protein concentration. The decay times were (3.0 ± 0.7 SEM) × 10^3^ min, (1.1 ± 0.3 SEM) × 10^3^ min, and (0.38 ± 0.15 SEM) × 10^3^ min for the incubations at 24, 28 and 32 μM, respectively. At 20 μM, the decrease in τ_A488_ was so slow that it was not possible to fit a single-exponential decay curve.

**Figure 5:**
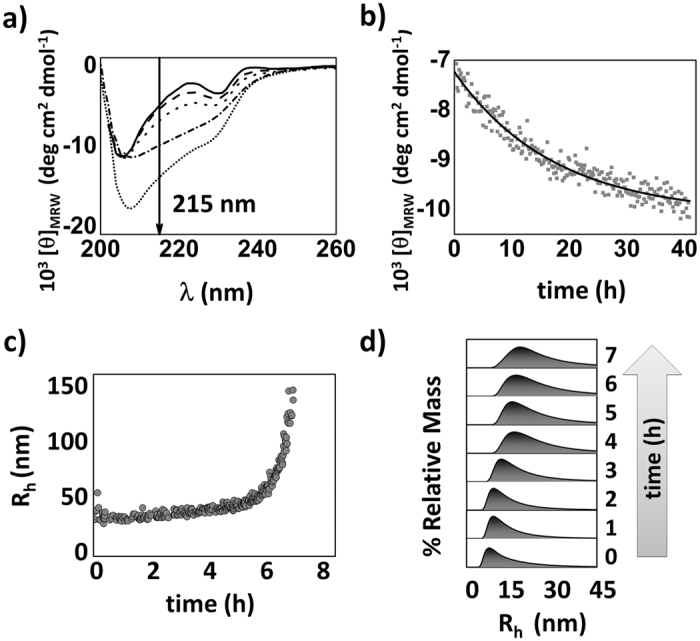
Bulk experiments with N47A-SH3-DA incubated at 32 μM. (**a**) Far-UV CD spectra at 20 °C (solid line), at 37 °C at “time zero” (dashed line), and after 12 h (dotted line), 48 h (dash-dotted line), and 45 days (short dashed line) of incubation. (**b**) Time evolution of the molar ellipticity measured at 215 nm. (**c**) Average R_h_ and (**d**) size distributions as a function of the incubation time obtained from the DLS experiments.

The molecular information obtained via SMF-PIE and FLIM imaging is unique compared with that obtained from conventional bulk techniques used in amyloid aggregation studies^7^. However, we also compared these results with the results obtained using conventional techniques to link the molecular-level observations with the macroscopic-level observations. Specifically, we performed far-UV circular dichroism (CD) and dynamic light scattering (DLS) experiments of labelled and unlabelled N47A-SH3 under aggregation conditions. Using far-UV CD, we monitored the negative molar ellipticity at 215 nm (Fig. 5a) as an estimate of the rate of β-sheet formation^21^ and, hence, the rate of formation of structured amyloid aggregates. The rate of β-sheet formation for 32 μM N47A-SH3-DA incubated at 37 °C exhibited an apparent decay constant, *k*_215_, of (9.6 ± 0.1 SEM) × 10^−4^ min^−1^ (Fig. 5b). This rate constant is on the same order as the apparent decay constant for the decrease in τ_A488_ in the oligomers obtained by FLIM (9.1 × 10^−4^ min^−1^ at 28 μM and 20.0 × 10^−4^ min^−1^ at 32 μM). Importantly, this result confirms the correlation between the formation of compact and, hence, high-FRET oligomers and the formation of β-sheet-rich species. Interestingly, the values of the apparent rate constants for the formation of type **2** oligomers, which were obtained from SMF-PIE, ranged from (6.1 × 10^3^) to (11.6 × 10^3^) min^−1^. These rate constants were slightly higher than the rate constants obtained using the FLIM-PIE and far-UV CD techniques. This result was obtained because we were able to specifically focus on individual type **2** events and discard the contributions from other species using SMF-PIE. This information is inaccessible using ensemble techniques; thus, the rate constant values obtained using FLIM and far-UV CD techniques also contain the contributions of other species (monomers, low FRET type **1** oligomers, etc.). The contributions of these species may cause an apparent slowing of the reaction.

Moreover, the DLS kinetic experiments provided information about the change in the oligomer size distribution during the aggregation of the N47A-SH3-DA mixture at 32 μM. The initial hydrodynamic radius (R_h_) value was approximately 30 nm, which was significantly larger than the estimated radius of the compact N47A-SH3 domain monomer (1.6 nm)^20^,^29^, indicating the rapid formation of type **1** aggregates at time 0^22^. The values of R_h_ and the size distributions (Fig. 5c and d) displayed slow growth in the initial hours (up to 6 h) but cooperative and rapid growth thereafter. These features are consistent with type **1** and **2** oligomers sharing similar sizes but growth only occurring when type **3** aggregates are formed.

These same bulk techniques also allowed us to compare the aggregation process of the unlabelled N47A-SH3 variant. Far-UV CD and DLS experiments of the unlabelled N47A-SH3 revealed features similar to those of the labelled protein (Supplementary Figs S6 and S7), although with generally slower aggregation kinetics. The enhancement of the aggregation rate caused by the fluorophores is driven by the aromaticity introduced by the dye moieties as previously shown through the introduction of aromatic amino acids into amyloid-aggregating proteins^24^.

### Oligomer toxicity

**Figure 6:**
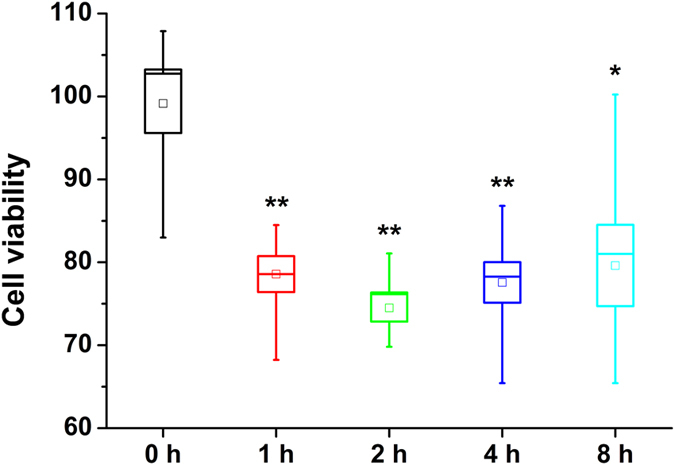
Cell viability assays of 32 μM N47A-SH3-DA oligomers. Aliquots collected after different incubation times (0, 1, 2, 4, and 8 h) were added to cultured 143B cells for 24 h. Cell proliferation was tested and compared with the untreated control cells. The boxes indicate the average values ± 1 SEM, and the whiskers indicate the minimum and maximum values of all repetitions. Asterisks indicate differences from the untreated control cells with 95% (*) or 99% confidence (**).

We performed a cell viability experiment using 143B cells to test the toxicity of the different types of the detected oligomers. Oligomers collected at different times of N47A-SH3-DA incubation were added to the cell cultures for 24 h, and cell proliferation was measured. Figure 6 shows that the type **1** oligomers, which dynamically formed at time 0, were essentially nontoxic. Once the type **2** oligomers formed, cell viability decreased, which confirms that this type of aggregate contained the most cytotoxic species. We did not observe a statistically significant difference in cytotoxicity at later times when the type **3** oligomers were detected. Similar trends in cytotoxicity were observed for the oligomers of unlabelled N47A-SH3 (Supplementary Fig. S8), considering the slower aggregation of this variant.

## Discussion

The primary goal of this very active field of multidisciplinary research is to provide insights on amyloid fibrillation mechanisms, particularly those associated with disease-related protein aggregation. However, conventional biophysical techniques are primarily limited to examinations of the entire oligomerization process that leads to the formation of amyloid fibers. We exploited the compatibility of SMF techniques with low concentrations to more closely observe the aggregation process. In particular, we exploited the superior features of advanced dual-colour PIE schemes in SMF and FLIM imaging experiments for improved characterization of the intra-oligomer FRET efficiency because every oligomer is probed individually for size, *E*, and τ_A488_. The use of the fluorescence lifetime of the donor dye, τ_A488_, provides an orthogonal assessment of the FRET efficiency and permits the unique correction of the oligomer size based on the quenching caused by the fluorescence of the donor dyes. This advanced characterization of the oligomers allowed us to unequivocally detect three different types of aggregates, **1**, **2**, and **3** (Fig. 2b), in the aggregation of the N47A-SH3 domain. By focusing our study on an SH3 domain, we used an excellent model to understand amyloid aggregation^19^ and formulate general hypotheses of the fibrillation mechanism.

**Figure 7:**
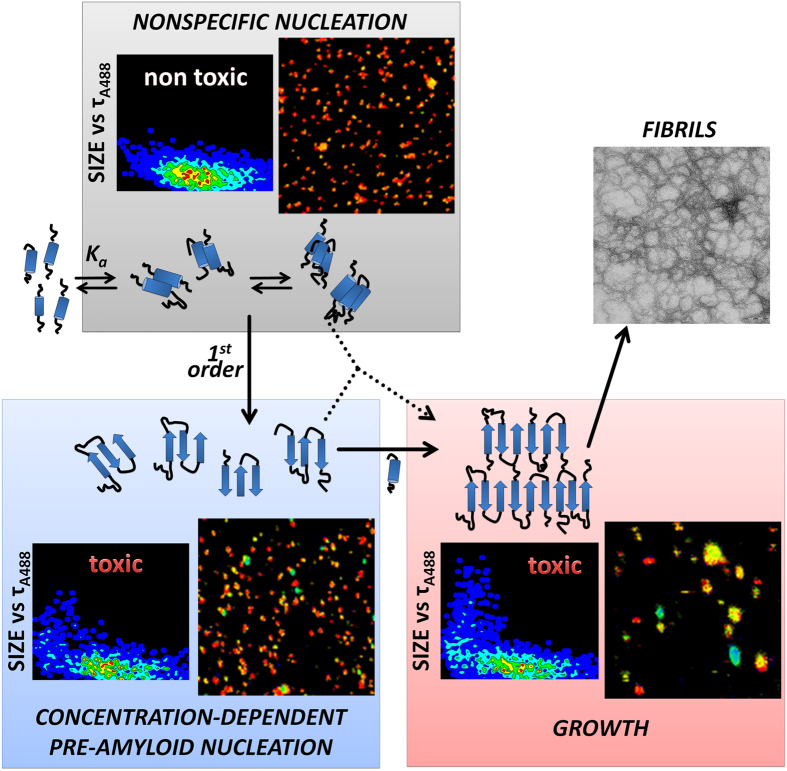
Proposed model of the two-step nucleation mechanism for amyloid aggregation, which is correlated with the supporting single-molecule (SMF-PIE and FLIM-PIE) evidence.

Recently, Knowles and collaborators used *in silico* experiments and reported the essential roles of nonspecific interactions in promoting amyloidogenic aggregation at low concentrations^2^. In this two-step nucleation model for amyloid aggregation at low concentrations, the first step involves the formation of contacts with early aggregates that will act as a scaffold for the second amyloid-prone nucleation step. Our results provide insights on the molecular conversions and growth steps that occur during the macroscopic lag phase and represent direct experimental evidence of this two-step nucleation mechanism. All of our experimental evidence indicates the need for an initial hydrophobically driven aggregation step for aggregation at low concentrations and the subsequent formation of the larger compact structures observed in the second nucleation step by a first-order conformational rearrangement (Fig. 7). The second type of nuclei exhibit amyloidogenic features and cytotoxicity. These important findings provide direct experimental evidence of the mechanisms by which amyloid fibrils form and suggest potential targets for the inhibition and reduction of the toxic effects of disease-related aggregating proteins.

## Methods

### Sample preparation and incubation

The N47A-SH3 mutant was purified and labelled using maleimide-reactive forms of the fluorophores ATTO 488 (A488) and ATTO 647 N (A647N, both from ATTO-Tec GmbH, Germany) as described elsewhere^22^. For the SMF, FLIM, and bulk experiments, equimolar mixtures of N47A-SH3 labelled with A488 and with 647 N were incubated at 37 °C in the aggregation buffer (0.1 M glycine and 0.1 M NaCl, pH 3.2) at total protein concentrations of 20, 24, 28, 32, and 44 μM. Aliquots of the incubation mixtures were collected at different times, snap-frozen, and stored at −42 °C until use. Three independent incubations were performed at each concentration.

### SMF-PIE methods and data analysis

Single-molecule fluorescence fluctuation traces were collected using a time-resolved confocal MicroTime 200 fluorescence microscope system (PicoQuant GmbH, Germany). A detailed description of the instrument can be found in the SI; briefly, it consisted of a SMF confocal microscope equipped with an excitation unit containing two spatially overlapped pulsed lasers (470 nm, LDH-P-C-470, and 635 nm, LDH-P-635, both from PicoQuant) that were alternated on a nanosecond time scale to achieve PIE^30^. The collected fluorescence was separated into two detection channels using a 600 DCXR dichroic beam splitter (AHF/Chroma, Germany) and directed to two avalanche photodiodes (APD) (SPCM-AQR-14, Perkin-Elmer Optoelectronics).

The data were analyzed using custom-coded scripts in SymPhoTime 32 (PicoQuant). The selection and counting of single-molecule oligomers in the coincident and FRET events were considered when a burst was simultaneously detected in the donor and directly excited acceptor traces. The burst-wise FRET efficiency (*E*), τ_A488_, and the apparent oligomer size were obtained for each oligomer event (see SI for details). We developed a novel correction for the oligomer size based on the accessibility of τ_A488_. For those oligomers in which τ_A488_ < τ_0_ (4.0 ns, the value for the unquenched A488 dye), the fluorescence intensity of the donor burst, F_A488_, was corrected by the factor τ_0_/τ_A488_. This correction takes into account the decrease in the emission intensity of A488 on the oligomers caused by FRET or any other dynamic quenching process. This correction was developed and employed here for the first time. Hence, the apparent oligomer size was estimated as follows:


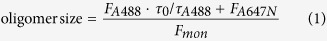


where F_A647N_ is the intensity of the directly excited A647N and F_mon_ is the average intensity of the monomer.

### FLIM-PIE methods and data analysis

FLIM-PIE images were collected using the same instrument described for SMF-PIE measurements. The aliquots were diluted 100,000–120,000-fold and deposited on glass slides that had been previously cleaned and treated for single-molecule imaging. The FLIM analyses were performed with SymPhoTime 32 by fitting a single exponential decay function to the photon decay trace in each pixel of the image. The localization of oligomers and extraction of the average τ_A488_ within the oligomers were performed using home-coded scripts in *Fiji is just ImageJ*^31^. Basically, pixels of oligomers were considered when they surpassed a certain intensity threshold in all three images simultaneously: A488, FRET, and A647N. Further details can be found in the SI.

### Ensemble biophysical techniques

Far-UV CD experiments were performed using a Jasco J-715 spectropolarimeter (JASCO, Japan). For the kinetic experiments, changes in the CD signal were monitored at 215 nm in a sample incubated in a 0.1-cm path length cuvette. For the dynamic light scattering (DLS) measurements, we used a DynaPro MS-X (Wyatt Technology Corp., Santa Barbara, CA, USA) equipped with a Peltier element to control the temperature of the sample. The samples were centrifuged at 14,000 rpm for 30 min to remove preexisting fibrillar forms.

### Cell viability assays

The impact of protein aggregation on the viability of human osteosarcoma 143B cells (see SI for details on the cell line) was studied using the CellTiter Blue™ viability assay (Promega). Cells were plated in quadruplicate on black, cell culture-treated 96-well optical flat-bottom plates at a density of 1.0 × 10^3^ cells/well. After 48 h of cell culture, 20-μL aliquots of the incubated samples (either unlabelled N47A-SH3 or labelled N47A-SH3-DA) were added directly to the wells. After the cells were incubated with the amyloid oligomers for 24 h, 20% v/v CellTiter-Blue™ reagent was added to the wells and incubated for 2 h at 37 °C. Fluorescence was read in a Glomax^®^-Multidetection System (Promega). Untreated cell controls and wells with reagents only served as background controls and were analyzed together with the treated cells. At least seven independent repetitions of each data point were performed. Statistical analyses of the cell viability of each population were compared against the 100% value of the untreated control cells using the Wilcoxon signed-rank test (in Origin 8.5, OriginLab Corp., MA, USA) to avoid the requirement for normally distributed populations.

## Additional Information

**How to cite this article**: Castello, F. *et al*. Two-Step Amyloid Aggregation: Sequential Lag Phase Intermediates. *Sci. Rep.* **7**, 40065; doi: 10.1038/srep40065 (2017).

**Publisher's note:** Springer Nature remains neutral with regard to jurisdictional claims in published maps and institutional affiliations.

## Supplementary Material

Supplementary Information
