# Supplementary material for: Two-Step Amyloid Aggregation: Sequential Lag Phase Intermediates
Source: Sci Rep. 2017 Jan 9;7:40065. doi: 10.1038/srep40065 (PMC5220338; doi:10.1038/srep40065)
Supplement: Supplementary Information [file srep40065-s1.pdf]

SUPPLEMENTARY INFORMATION

Two-Step Amyloid Aggregation: Sequential Lag Phase Intermediates

Fabio Castello,[1] *Jose M. Paredes,*[1] *Maria J. Ruedas-Rama,*[1,2] *Miguel Martin,*[2] *Mar Roldan,*[2] *Salvador Casares,*[*,3] and *Angel Orte*[*,1,2]

[1] Dept. of Physical Chemistry, Faculty of Pharmacy, University of Granada, Cartuja Campus, 18071, Granada (Spain)

[2] GENYO, Pfizer-University of Granada-Junta de Andalucia Centre for Genomics and Oncological Research. Avda Ilustracion 114. PTS. 18016, Granada (Spain)

[3] Dept. of Physical Chemistry, Faculty of Sciences, University of Granada, Fuentenueva Campus, 18071, Granada (Spain)

* **Corresponding author**: Angel Orte. E-mail: angelort@ugr.es, Tel. +34 958243825. Salvador Casares. E-mail: scasares@ugr.es

TABLE OF CONTENTS

S1 Transmission Electron Microscopy (TEM) S3
Supplementary Fig. S1 S3
S2 Single molecule fluorescence spectroscopy with dual-color pulsed interleaved excitation (SMF-PIE): Instrumentation, methods and analysis S4
Supplementary Fig. S2 S7
Supplementary Fig. S3 S8
Supplementary Table S1 S9
S3 Fluorescence lifetime imaging microscopy with dual-color pulsed interleaved excitation (FLIM-PIE) experiments S9
Supplementary Fig. S4 S10
Supplementary Fig. S5 S11-13
S4 Far-UV circular dichroism (far-UV CD) S14
Supplementary Fig. S6 S14
S5 Dynamic light scattering (DLS) S15
Supplementary Fig. S7 S16
S6 Cell viability experiments S16
Supplementary Fig. S8 S17
SI References S18

S1 Transmission Electron Microscopy (TEM)

TEM measurements were performed using a Libra 120 Plus transmission electron microscope (Carl Zeiss SMT, Germany) operated at 120 kV and equipped with a LaB6 filament and an SSCCD 2 k × 2 k direct coupling camera.

The protein samples (N47A-SH3-DA) were diluted in a buffer that promoted aggregation (0.10 M NaCl and 0.10 M Gly, pH 3.2) and were incubated at 37 °C for 45 days. Ten microliter aliquots were removed at appropriate times during the incubation of the aggregation sample, and they were rapidly frozen in liquid nitrogen to stop the aggregation. Prior to the TEM measurements, these aliquots were deposited on Formvar 300-mesh copper grids (ANAME, Spain). After a 5 minute incubation, the copper grids were washed twice with deionized water and negatively stained with a 1% (w/v) uranyl acetate solution for 1 minute. The excess stain was removed with tissue paper. Finally, the samples were dried and used for the TEM analysis.

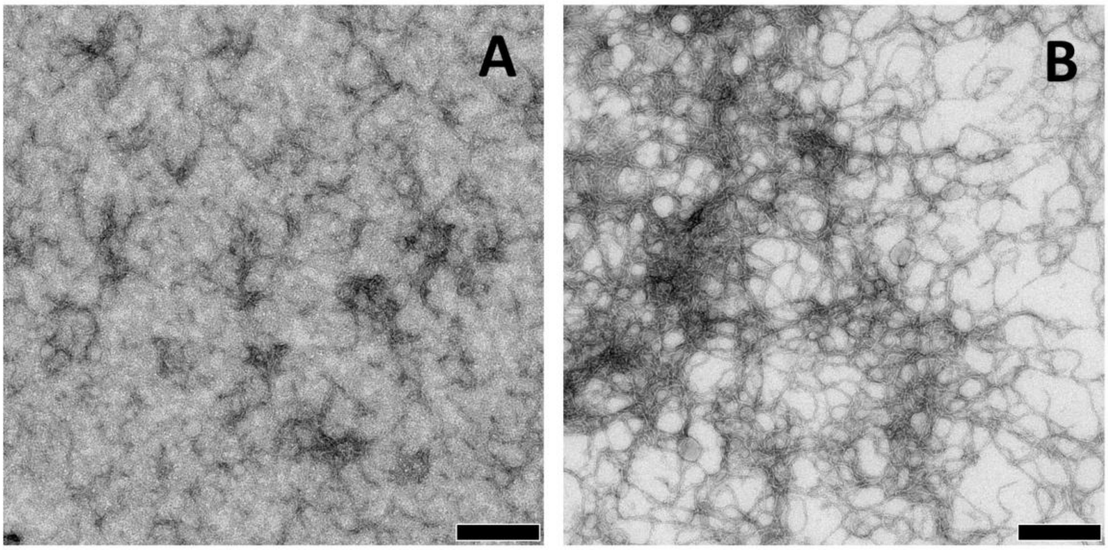

Supplementary Figure S1. TEM images of amyloid fibrils of N47A-SH3-DA. The fractions used for TEM experiments were collected after the samples were incubated for 8 hours (A) and 45 days (B) under aggregation conditions at a total protein concentration of 32 µM. Black scale bar = 200 nm.

S2 Single molecule fluorescence spectroscopy with dual-color pulsed interleaved excitation (SMF-PIE): Instrumentation, methods and analysis

S2.1 Instrumentation. SMF-PIE experiments were performed using a MicroTime 200 time-resolved fluorescence microscope (PicoQuant GmbH, Germany). Excitation was achieved through two spatially overlapped pulsed lasers (PicoQuant LDH-P-C-470 system at 470 nm and a PicoQuant LDH-P-635 system at 635 nm) that alternated on a nanosecond time scale. The overall excitation pulse frequency was 20 MHz. The 635 nm laser was delayed by 24 ns (ORTEC DB463 delay box, Ametek, USA) to achieve pulsed interleaved excitation (PIE) [1]. The excitation powers were 286 μW for the 470 nm laser and 22.7 μW for the 635 nm laser. The laser outputs were focused to the back port of an inverted microscope (Olympus IX-71, Japan) via fiber coupling after passing the main dichroic mirror, which was a custom-made, dual-band dichroic mirror (AHF/Chroma), allowing the simultaneous excitation of the two lasers and collection of the two fluorescence bands. The beams were focused through a high numerical aperture oil-immersion objective (Plan Achromat 100x/1.40 NA, Olympus, Japan) into ~ 60 μL of sample solution placed on a microscope cover glass (Thermo-Scientific, Menzel-Gläser, Germany), with the lasers focused 10 μm into the solution. The fluorescent photons were collected by the same objective and imaged onto a 75 μm pinhole (Melles Griot, USA). The transmitted light was separated by a 600 dcxr dichroic beam splitter (AHF/Chroma) after it passed through the aperture, filtered into the donor (520/35, Omega Filters) and acceptor (685/70, Omega Filters) channels, and finally directed to two avalanche photodiodes (SPCM-AQR-14, Perkin-Elmer Optoelectronics, USA).

Our setup allows time-tagged time-resolved (TTTR) measurements that tag every single photon event in terms of the macro-time, micro-time (to reconstruct fluorescence decay traces), and detector number.

S2.2 SMF-PIE data analysis. The data were analyzed using home-coded scripts in SymPhoTime 32 (PicoQuant). The fluorescence signals of the donor (F_{A488}) FRET (F_{FRET}), and directly excited A647N (F_{A647N}) were separated by choosing adequate time windows and applying temporal filters (Fig. 1, main text). These fluorescence intensities were corrected for the corresponding background of the buffer in each channel and time window. A value of 15 kHz was set for F_{A488} and F_{A647N} to select and count single-molecule bursts, which allowed us to distinguish a burst originating from a

fluorescent molecule in the confocal volume from the background signal. The coincidence criterion of simultaneous detection of a burst in both channels (F_{A488} and F_{A647N}) [2] was applied to detect the protein aggregates. Importantly, the direct acceptor excitation in PIE mode permitted us to remove contributions from the 'zero-peak' present in conventional single-molecule FRET measurements [3]. SMF-PIE is a powerful, dual-color method that is capable of extracting a variety of parameters that can be correlated for each individual molecule event to construct multi-dimensional population histograms (Fig. 1, main text). For each individual aggregate detected, we estimated the (burst-wise-obtained) FRET efficiency, *E*, the fluorescence lifetime of donor, τ_{A488}, and the apparent oligomer size.

The burst-wise FRET efficiency, *E*, was obtained with F_{A488} and F_{FRET} (emissions detected at the 470-nm excitation time window) using eq. S1. The FRET signal must be corrected for the spectral crosstalk of the donor dye in the acceptor detection channel (β = 0.0174), the fraction of direct excitation of the acceptor by the donor laser (α = 0.017), and detection correction factor (γ = 0.95) [4-5].

$$E = \frac{F_{FRET} - \alpha F_{A647N} - \beta F_{A488}}{F_{FRET} - \alpha F_{A647N} - \beta F_{A488} + \gamma F_{A488}} \qquad \text{(S1)}$$

For each aggregate burst, the fluorescence lifetime of the donor, τ_{A488}, was obtained by fitting a single exponential decay function to the photon decay trace using iterative deconvolution with a simulated instrument response function (IRF). The maximum likelihood estimator was employed as a criterion for optimizing the fits because this criterion works better than others in cases with low photon counts. The value of τ_{A488} represents an orthogonal estimation of the FRET efficiency through the quenching caused by the A488 fluorophore because $E = 1 - \tau_{A488}/\tau_0$, where τ_0 is the fluorescence lifetime of the donor in the absence of the acceptor, which is 4.0 ns for A488 [6]. Hence, τ_{A488} involves an estimation of the FRET efficiency independent from the burst intensities and is a much more reliable approach than the estimation of both *E* and oligomer size from the same burst-wise intensity, a method that was previously employed to study α-synuclein oligomers [7-9].

Finally, we used the total intensity of the directly detected A488 and A647N (F_{A488} and F_{A647N}) divided by the average intensity of a monomer to estimate the apparent oligomer size of the aggregates detected. This simple method has been used previously

[10-11]; however, it presents an important problem when there is intra-oligomer FRET: the quenching caused by the donor's intensity because FRET can cause an underestimation of the size. Cremades and colleagues corrected this issue using the burst-wise FRET efficiency *E* as the correction factor based on the donor's emission intensity [8-9]. This method is not free of problems because the FRET efficiency and the oligomer size are obtained with the same bursts, thus causing clear correlations. SMF-PIE has two main advantages when correcting this issue: 1) it separates the donor excitation and the acceptor direct excitation in the different time windows to avoid crosstalk and 2) the fluorescence lifetime of the dyes is accessible as a measurement of all the quenching processes that may result in a decrease of the fluorescence emission intensity. Based on these advantages, we developed a modification of the oligomer size estimate using two different equations that depend on the τ_{A488} value by considering the quenching caused by FRET and other processes.

For molecules exhibiting $\tau_{A488} \geq \tau_0$,[6] the quenching and FRET processes are negligible. For these molecules, the apparent oligomer size of each individual event was calculated in the conventional way as the total intensity of the burst by considering both channels and dividing by the average fluorescence intensity of a monomer (eq. S2) [10-11]:

$$\text{Oligomer size} = \left(\frac{F_{A488} + F_{A647N}}{F_{mon}} \right) \qquad \text{(S2)}$$

For molecules exhibiting $\tau_{A488} \leq \tau_0$, the overall intensity in the A488 channel was corrected by the amount of quenching using a factor that accounted for the lifetime ratio, τ_0/τ_{A488} (eq. S3):

$$\text{Oligomer size} = \left(\frac{F_{A488}\frac{\tau_0}{\tau_{A488}} + F_{A647N}}{F_{mon}} \right) \qquad \text{(S3)}$$

In these equations, F_{mon} is the average fluorescence emission intensity of a monomer. F_{mon} was obtained from 17 different SMF-PIE experiments performed between 75 and 1000 pM of total protein concentration. We obtained the average intensity of the non-coincident bursts in the A488 and A647N channels, as non-coincident bursts are mainly caused by monomers. These average intensities were 29.7 kHz for A488 and 31.9 kHz for A647N. The intensity for the monomer, F_{mon}, that we used in eqs. S2 and S3 was the average of those two values: 30.8 kHz, for simplifying the equations.

S2.3 SMF-PIE correlograms. The SMF-PIE approach allows us to construct multi-dimensional histograms using the different parameters obtained from single-molecule events. Supplementary Figure S2 shows the single-molecule correlograms of burst-wise FRET efficiency, *E*, vs. the donor fluorescence lifetime, τ_{A488}, from aliquots of the incubation of N47A-SH3-DA at different total protein concentrations.

However, the most interesting correlograms were those that correlated the apparent oligomer size vs. τ_{A488}. Supplementary Figure S3 shows these correlograms from aliquots of N47A-SH3-DA samples that were incubated at different total concentrations and incubation times.

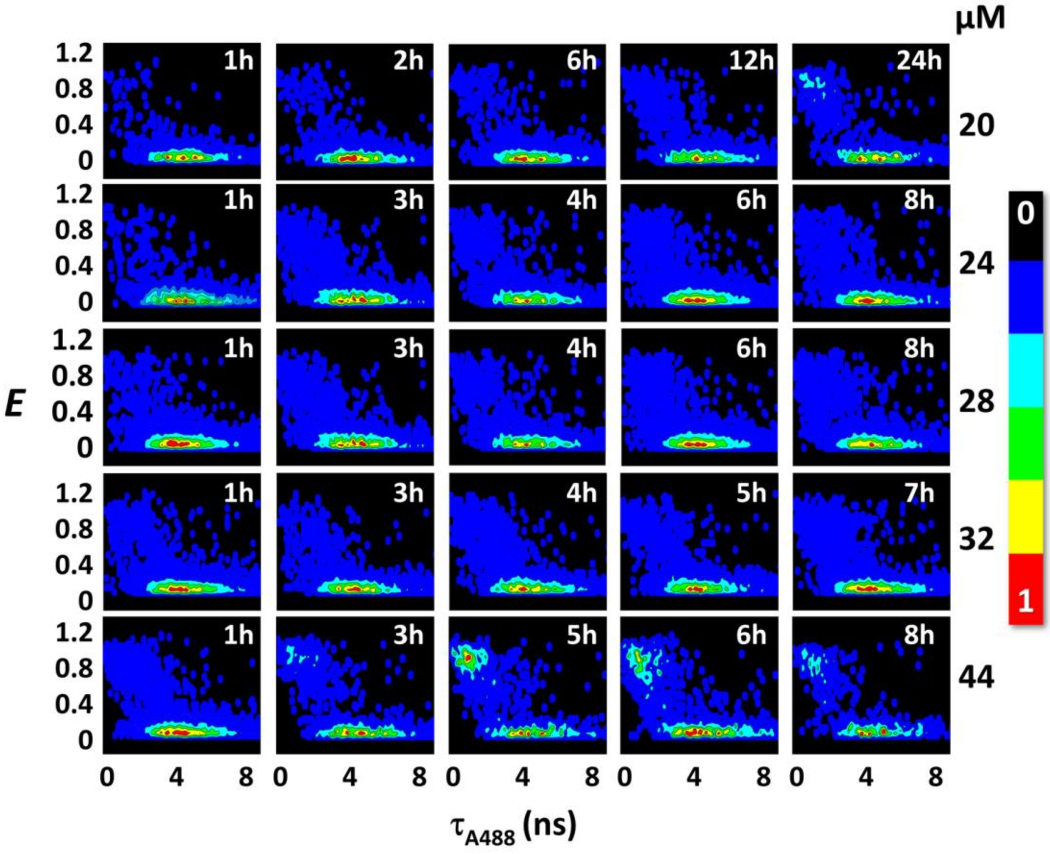

Supplementary Figure S2. Single-molecule correlograms of the FRET efficiency (*E*) vs. the donor fluorescence lifetime (τ_{A488}) of N47A-SH3-DA incubated in the aggregation buffer at 37 °C at total protein concentrations of 20, 24, 28, 32, and 44 μM. The graphs show the correlograms of aliquots collected at different incubation times.

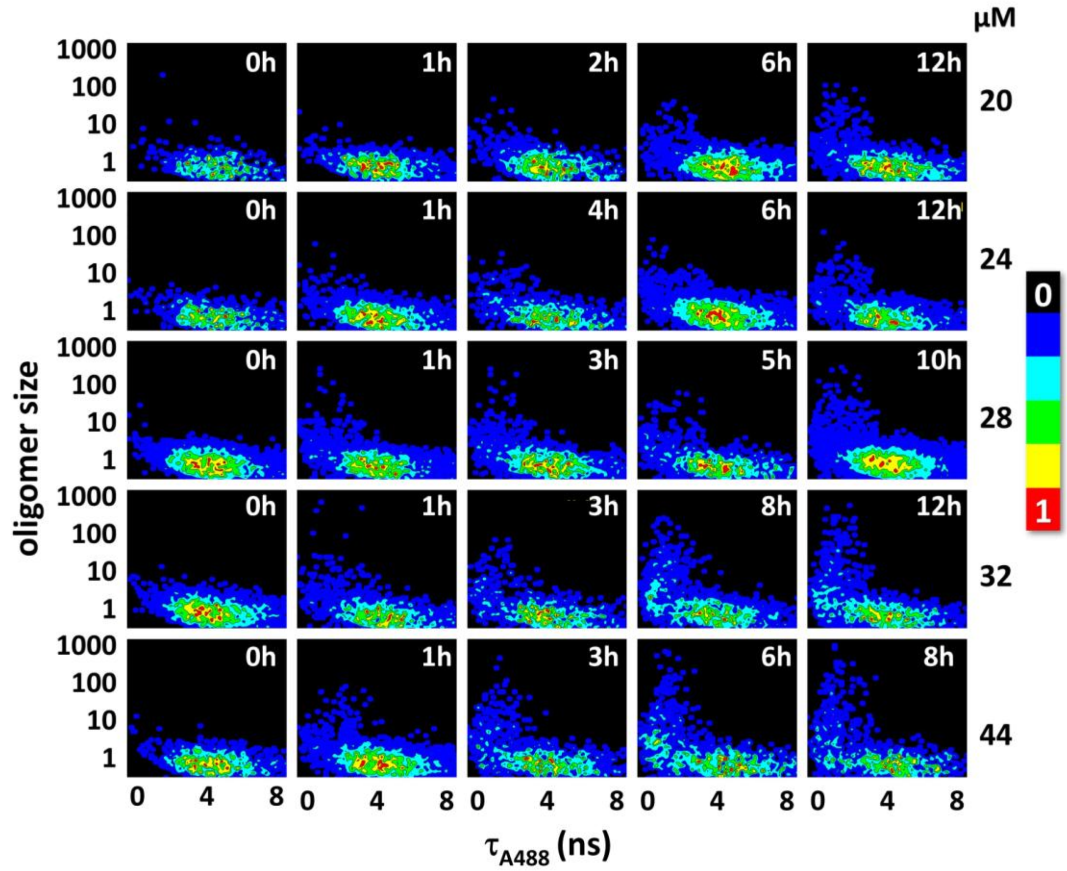

Supplementary Figure S3. Single molecule correlograms of the oligomer size vs. τ_{A488}. Aliquots of N47A-SH3-DA samples were collected at different times during incubation of total protein concentrations of 20, 24, 28, 32, and 44 μM in the aggregation buffer at 37 °C.

S2.4 Kinetic study of the SMF-PIE results: Initial rates of oligomer formation and disappearance. We performed a thorough study of the kinetics of the N47A-SH3-DA aggregation using the SMF oligomer size vs. τ_{A488} correlograms. By defining the appropriate threshold areas, the relative contributions of the three types of oligomers were quantified (Fig. 2B, main text). We applied the following corrections in the amounts of each type of oligomer: 1) removing the contribution of chance coincident events from type 1 oligomers; and 2) correcting the number of small oligomers by the statistical probability of finding oligomers composed of just a single type of dye (either A488 or A647N). This method allowed us to uniquely follow the kinetics of the three types of aggregates and infer mechanistic conclusions. We studied the time evolution of these contributions (Figs. 2 and 3 in main text) and employed the initial rate method to quantify the kinetics and order of reaction of the formation of types **2** and **3** oligomers, as well as the disappearance of the type **1** aggregates. By fitting the experimental data to single exponential decay functions (Fig. 3A, main text), we obtained the initial rate as

the derivative of the relative population of oligomer with time, at time = 0 ($v_0 = |d[C]/dt|_{t=0}$, where [C] is the concentration of each type of oligomer, **1**, **2**, or **3**) [12]. The apparent order of the reaction was obtained from the slope of the logarithmic plot of the initial rates versus the total protein concentration (Fig. 3B in main text). The initial rates and the apparent values of the reaction order are compiled in Supplementary Table S1.

Supplementary Table S1. Initial rates (absolute values) of the disappearance (type **1**) and formation (types **2** and **3**) of the different types of oligomers along with their corresponding apparent reaction order.

Oligomer type		Concentration of N47A-SH3-DA (µM)					Apparent reaction order
		44	32	28	24	20	
1	Initial rate (µM min^{-1})	0.51	0.33	0.28	0.12	0.045	3
2		0.11	0.081	0.065	0.051	0.022	1.8
3		0.36	0.23	0.18	0.055	0.032	3

S3 Fluorescence lifetime imaging microscopy with dual-color pulsed interleaved excitation (FLIM-PIE) experiments

S3.1 Instrumentation. FLIM images were recorded with the same MicroTime 200 fluorescence lifetime microscope system described above. The excitation powers were 6.64 µW for the 470 nm laser and 0.275 µW for the 635 nm laser. The microscope cover slides (Thermo Fisher Scientific, MENZEL-GLÄSER, Germany) were thoroughly cleaned using a previously described protocol [13]. After this procedure, the slides were stored in distilled water and, immediately prior to use, they were washed with ethanol and briefly air-dried. The laser beams were focused just above the surface of the slide and a wide area (15 µm^2) was raster-scanned with an x-y piezo-driven device (PI, Physik Instrumente, Germany) to set a region of interest (ROI) and image the ROI. The images were acquired with a 512×512 pixel resolution, a collection time of 0.60 ms/pixel, and a time resolution of 116 ps per channel in the single-photon timing scale. At least 10 images were collected from various areas for each sample.

The FLIM images were analyzed using the SymPhoTime 32 software package and a

similar protocol to the protocol used in the SMF-PIE traces but in a pixel-by-pixel fashion. The PIE excitation scheme allows dual-color excitation but is separated into two different time windows; therefore, simultaneous images of two different fluorophores can be reconstructed. We performed spatial pixel binning from 3×3 to 5×5 on the raw image to increase the number of photons per pixel. By selecting the time windows and the detection channels for the donor images and the FRET and acceptor images, the PIE scheme allowed for the one-step reconstruction of the donor dye FLIM image, FRET fluorescence image, and directly excited acceptor FLIM image (Supplementary Fig. S4). For the analysis of specific aggregates, e.g., the aggregates that showed similar τ_{A488} values, we selected those particles that were present in all three donors, FRET images, and acceptor images above a certain photon count threshold (Supplementary Fig. S4).

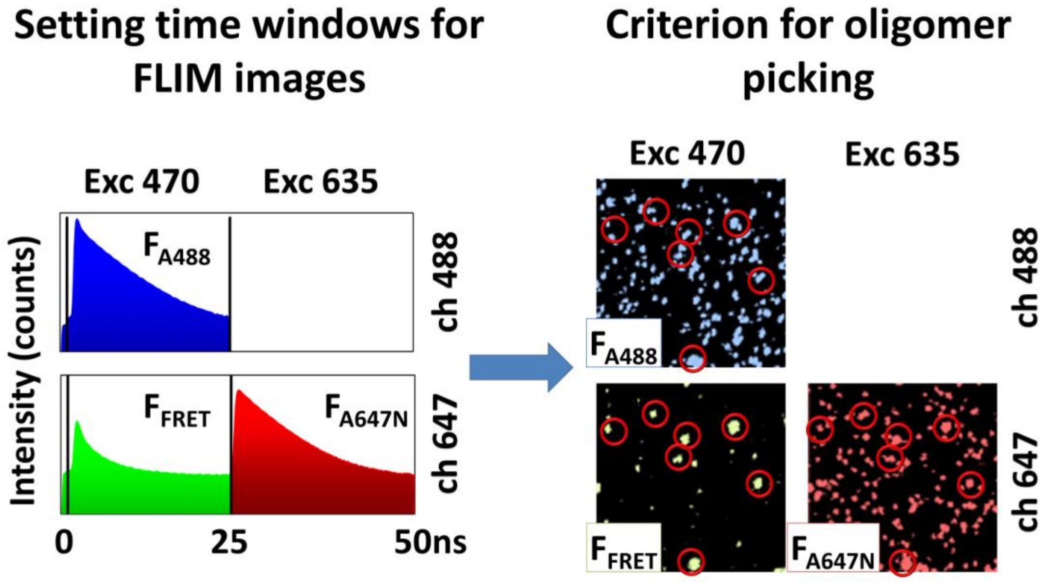

Supplementary Figure S4 Scheme of FLIM-PIE experiments. PIE scheme in FLIM permits the acquisition of three simultaneous images from a single scan: A488, FRET, and directly excited A647N.

The FLIM measurement delivers images with a decay curve in each pixel. For the τ_{A488} FLIM images, the decay curves in the individual pixel were fitted with a single exponential decay function by applying the maximum likelihood estimator (MLE) as criterion for goodness of fit. For the deconvolution analysis, an instrument response function (IRF) was reconstructed from the decay of a FLIM measurement performed at high concentration of sample (from 0.5 to 2 nM) that contained the same number of

time channels as the dataset to be analyzed. Only the pixels that surpassed a threshold of 5 counts at the maximum channel were analyzed to discard the background fluorescence. After the FLIM analysis, the images were displayed as pseudo-color plots depending on the τ_{A488} value (Supplementary Fig. S5). The fluorescence lifetime distributions of τ_{A488} shown in Fig. S5 are constructed only from pixels containing aggregates (coincident pixels in the three images: F_{A488}, F_{FRET}, and F_{A647N}).

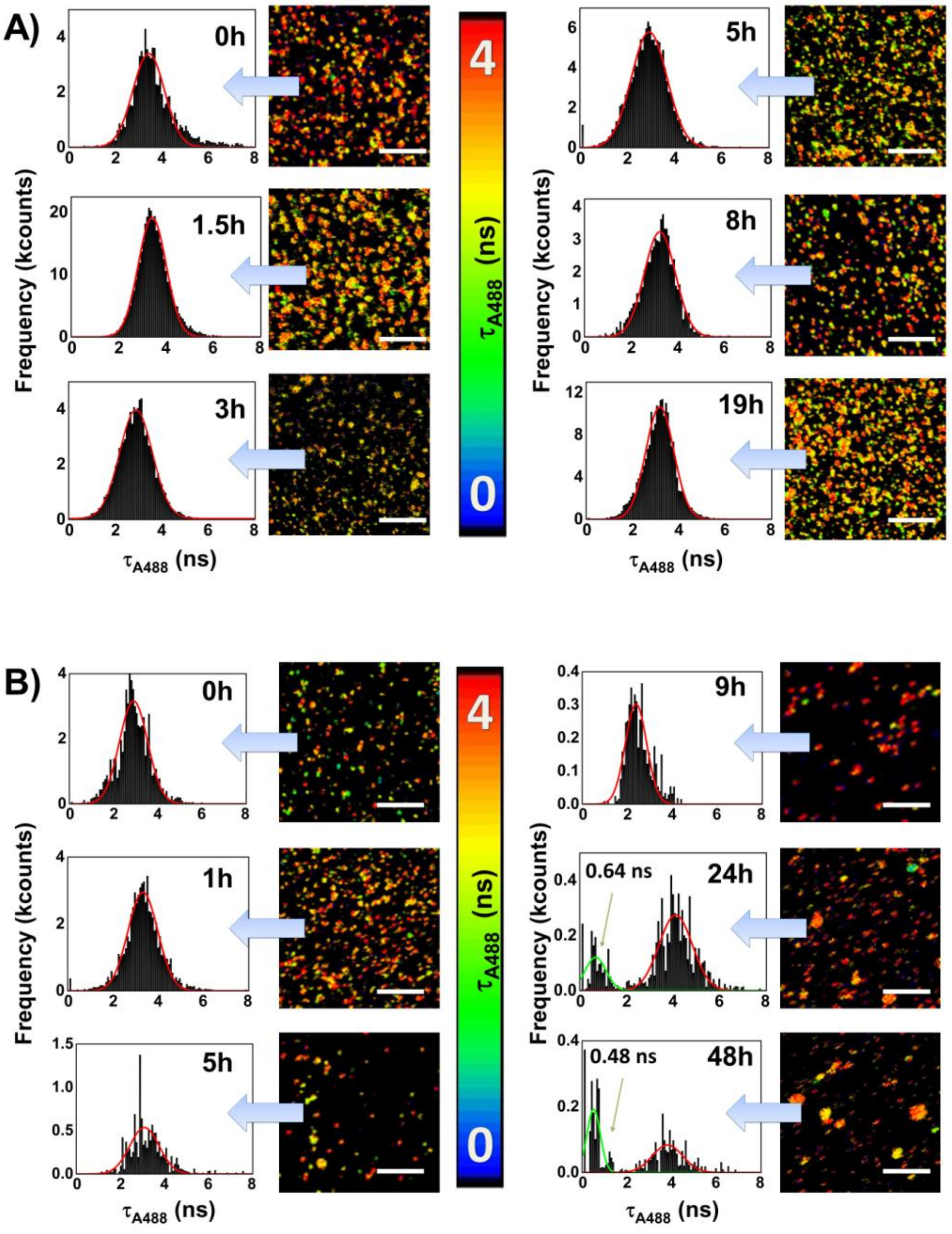

Supplementary Figure S5 A, B. Donor FLIM images and the corresponding lifetime distributions of 20 (**A**) and 24 (**B**) μM N47A-SH3-DA incubated in the aggregation buffer at 37 °C. White scale bars: 2.4 μm. Color scale: 0 to 4 ns.

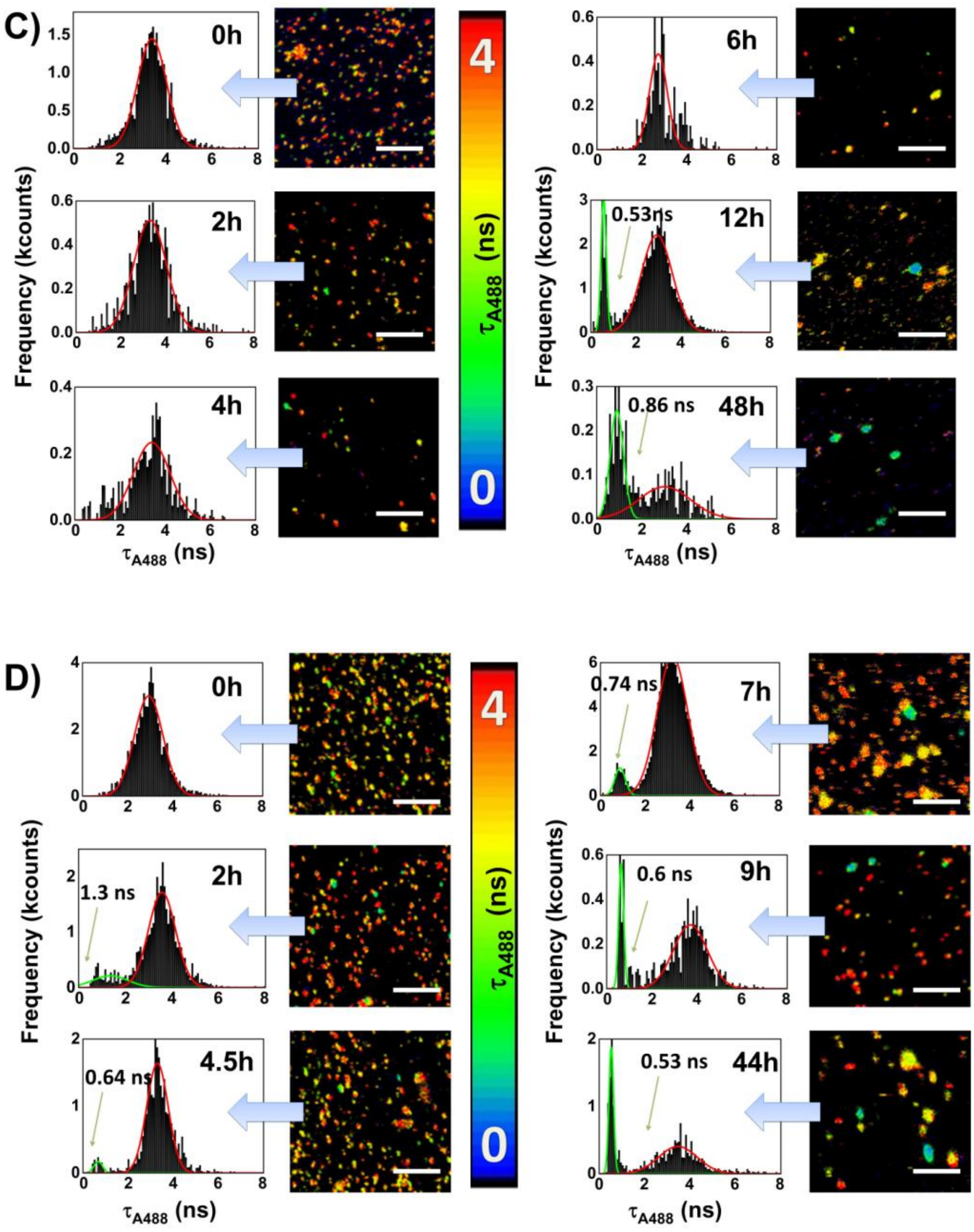

Supplementary Figure S5 C, D. Donor FLIM images and the corresponding lifetime distributions of 28 (C) and 32 (D) µM N47A-SH3-DA incubated in the aggregation buffer at 37 °C. White scale bars: 2.4 µm. Color scale: 0 to 4 ns.

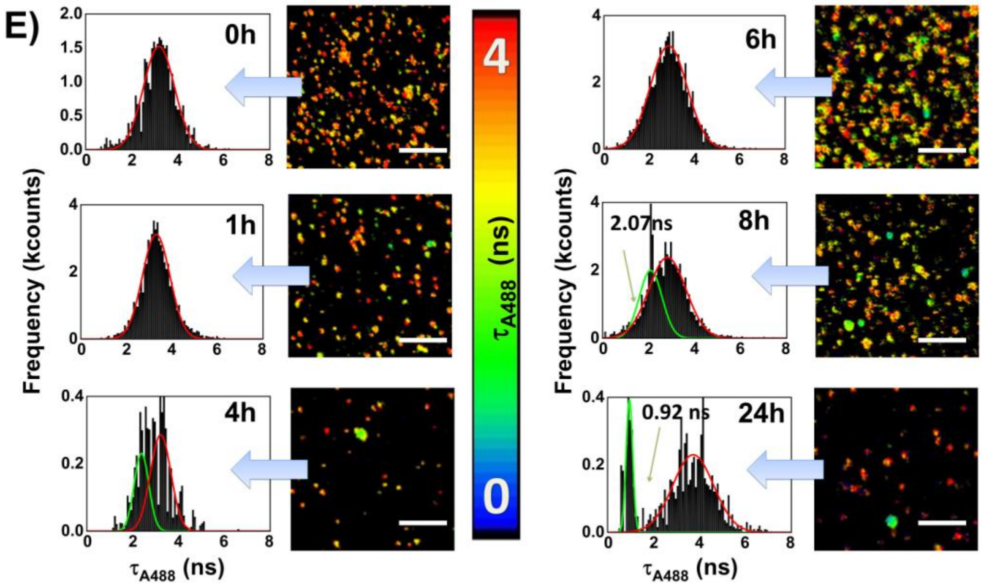

Supplementary Figure S5 E. Donor FLIM images and the corresponding lifetime distributions of 44 µM N47A-SH3-DA incubated in the aggregation buffer at 37 °C. White scale bars: 2.4 µm. Color scale: 0 to 4 ns.

S3.2 Kinetic studies using FLIM. A home-coded *Fiji is just ImageJ* [14] macro was used as an alternative analysis for the kinetic studies performed using FLIM data [14] to obtain the average τ_{A488} within the aggregates in the images. In detail, after exporting the output matrix data of the FLIM analysis, a Gaussian smoothing function was applied (s.d. = 1, in pixels). Then, the three channels (F_{A488}, F_{FRET}, and F_{A647N}) were semi-automatically segmented based on the pixel intensity using an isodata algorithm ('getAutoThreshold' method or 'make binary' function in Fiji and binary masks created, that is, 0 for background and 1 for oligomeric species). By multiplying each intensity-masked channel, we obtained a final image, which only contains the values for the coincident events. Finally, we multiplied the final masked images by the τ_{A488} to obtain a final image where each intensity-pixel was associated with the corresponding τ_{A488} (Fig. 4, main text). For the kinetic study, the average value of τ_{A488} in all the pixels containing oligomers was assessed.

S4 Far-UV circular dichroism (far-UV CD)

Far-UV CD spectra of N47A-SH3-DA and unlabeled N47A-SH3 were collected using a JASCO J-715 (JASCO, Japan) spectropolarimeter equipped with Peltier-driven temperature controller under a N_2 atmosphere (5 L/min). The parameters used in the measurements were as follows: 1 mm quartz cuvette; wavelength, 260 nm to 200 nm; response time, 1 sec; scan speed, 100 nm min^{-1}; bandwidth, 1 nm; and temperature, 37 °C. The acquired data were subtracted from a baseline scan of the buffer. We added 0.1 M *tris*(2-carboxyethyl)phosphine (TCEP) to the unlabeled samples to avoid undesired interactions with the free terminal cysteine in the protein. We followed the enhancement of the CD signal at 215 nm to examine the appearance of β-sheet structures [15]. Previous studies have monitored the changes in the N47A Spc-SH3 secondary structure by examining the CD signal at 215 nm during the time course of aggregation at 37 °C [16-17]. The rate of β-sheet formation was obtained by fitting this CD time trace to a single-exponential decay function, from which we obtained the apparent decay constant at 215 nm, k_{215}.

The results of the k_{215} value for the aggregation of the labeled sample (N47A-SH3-DA) are reported in the main text. Figure S6 shows the CD spectra and the time evolution of the 215 nm CD signal for the incubation of unlabeled N47A-SH3. The recovered k_{215} was $(2.7 \pm 0.1)\times10^{-4}$ min^{-1}, which was slightly less than the value obtained for the N47A-SH3-DA (see main text). The enhanced rate of the labeled construct is caused by the increase in hydrophobicity introduced by the dyes' moieties [18].

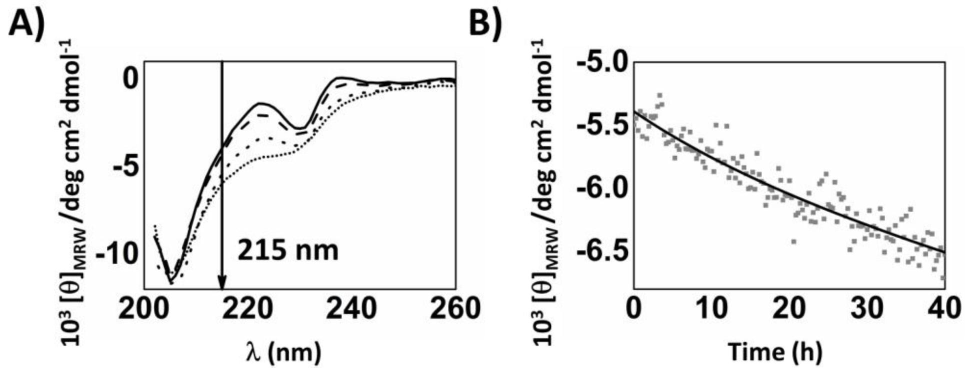

Supplementary Figure S6. Far-CD experiments of 32 μM N47A-SH3. A: Far-CD spectra of aggregating N47A-SH3. Solid line: N47A-SH3 at 20 °C, dashed line: N47A-SH3 at 37 °C, dotted line: N47A-SH3 after a 12 h incubation at 37 °C, short dotted line: N47A-SH3 after a 60 h incubation. B: CD kinetics at 215 nm of 32 μM N47A-SH3 incubated at 37 °C.

S5 Dynamic light scattering (DLS)

The DLS experiments were performed to provide information about the changes in the oligomer size distribution during the aggregation of the protein samples. DLS measurements were collected on a DynaPro MS-X instrument (Wyatt Technology Corporation, Santa Barbara, CA, USA) equipped with a Peltier element for temperature control of the sample. The samples were centrifuged at 14,000 rpm for 30 min to remove the preexisting fibrillar forms. We added 5 mM dithiothreitol (DTT) to the buffer solution to avoid possible interactions between the cysteine residues of the unlabeled N47A-SH3 samples.

The DLS results for the aggregation of the labeled sample (N47A-SH3-DA) are reported in the main text. The aggregation process of the 120 μM N47A-SH3 samples (Supplementary Fig. S7A) is characterized by the presence of two different species: smaller oligomers, with R_h values of approximately 1.7 nm, corresponding to a native monomer [19-21], and large oligomers, whose R_h increases as the incubation proceeds (Supplementary Fig. S7B). At a 60 μM protein concentration, the R_h value increases from the initial from 1.7 nm value, with a prolonged lag phase (Supplementary Fig. S7A). However, the sensitivity range of the DLS technique allowed us to perform a kinetic study of the growth of mainly the larger aggregates, whereas the presence of smaller aggregates can be masked. Indeed, the dependence of the intensity on the sixth power of the diameter of the particles causes larger species to dominate the intensity distribution [22].

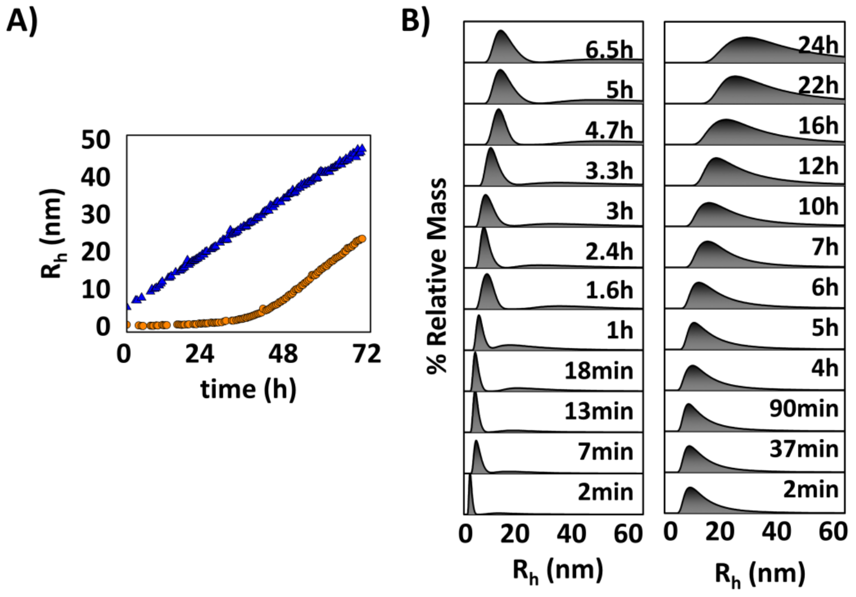

Supplementary Figure S7. DLS experiments of samples of N47A-SH3 incubated at 37 °C in the aggregation buffer. A: Kinetic experiments of 120 (blue diamond) and 60 μM (orange circle) N47A-SH3. B: Size growth of the two visible aggregates formed in the mass distribution of 120 μM N47A-SH3 as a function of the incubation time.

S6 Cell viability experiments

The human osteosarcoma cell line 143B was obtained from the American Type Culture Collection (ATCC; CRL-8303). The cells were cultured with high glucose Dulbecco's Modified Eagle's Medium (DMEM) containing Glutamax™-I (4 mM), sodium pyruvate (1 mM) (GIBCO, Life Sciences), 1% penicillin/streptomycin (GIBCO, Life Sciences), and 10% fetal bovine serum (FBS) (GIBCO, Life Sciences). The cells were subcultured every 3-4 days at a density of 2.4 or 5.6×10^3 cells/cm^2, and the media were refreshed the day before subculture.

The impact of protein aggregation on cell viability was studied using the CellTiter Blue™ viability assay (Promega). The cells were plated in quadruplicate in black, cell culture-treated 96-well optical flat bottom plates at a density of $1.0\text{x}10^3$ cells/well. After 48 h of cell culture, 20 μL aliquots of the protein samples (either unlabeled N47A-SH3 or labeled N47A-SH3-DA incubated under amyloidogenic conditions) were added directly to the wells. Aliquots of 32 μM N47A-SH3-DA that had been incubated in aggregation buffer at 37 °C were removed at 0, 1, 2, 4, and 8 h of incubation. For unlabeled N47A-SH3, aliquots were collected after 0, 2, 8, and 24 h of incubation because the kinetics of aggregation of the unlabeled variant are slower than the labeled

domains. After the cells were incubated with the amyloid oligomers for 24 h, 20% v/v CellTiter-Blue™ (Promega) reagent was added to the wells, incubated for 2 hours at 37 °C, and then the fluorescence was directly read at 525/580-640 nm in a Glomax®-Multidetection System (Promega). Untreated cell controls and wells with reagents only served as background controls and were run together with the treated cells. The absolute fluorescence was recorded in arbitrary units and subsequently the data were expressed as percentages relative to the untreated control cells. At least seven independent repetitions of each data point were performed.

Statistical analyses of the cell viability of the populations were compared to the 100% value of untreated control cells using the Wilcoxon signed-rank test (using Origin 8.5, OriginLab Corp., MA) to avoid the requirement for normally distributed populations.

The cell viability results showing the effect of the oligomers from the labeled sample (N47A-SH3-DA) are reported in the main text (Fig. 6). Supplementary Figure S8 shows that the oligomers formed by the unlabeled N47A-SH3 are again toxic after a sufficient incubation time for type **2** oligomers to appear. The toxicity profile is very similar to the labeled protein. This profile shows nontoxic species at incubation time 0 and appreciable toxicity that was significantly different from the untreated controls at 95% confidence at later incubation times.

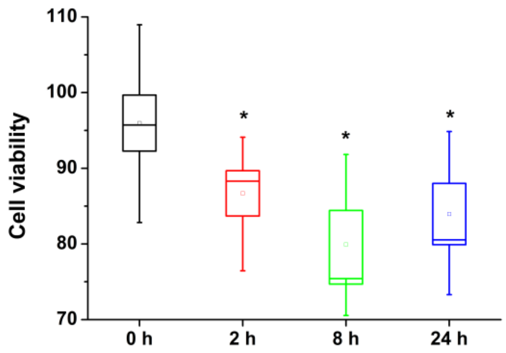

Supplementary Figure S8. Cell viability assays upon the addition of 32 μM N47A-SH3 samples that were incubated at 37 °C. Aliquots of the N47A-SH3 samples were collected after different incubation times and were added to the cultured 143B cells. Cell proliferation was tested and compared with the untreated control cells. Boxes indicate average values ± 1 standard error, whereas whiskers indicate the minimum and maximum values of all the repetitions. * Different from untreated control cells with 95% confidence.

SI References

1. Muller, B.K., Zaychikov, E., Brauchle, C. & Lamb, D.C. Pulsed interleaved excitation. *Biophys J* **89**, 3508-3522 (2005).
2. Orte, A., Clarke, R. & Klenerman, D. Single-molecule two-colour coincidence detection to probe biomolecular associations. *Biochem Soc Trans* **38**, 914-918 (2010).
3. Ruedas-Rama, M.J., Alvarez-Pez, J.M. & Orte, A. Solving single biomolecules by advanced fret-based single-molecule fluorescence techniques. *Biophys Rev Lett* **8**, 161-190 (2013).
4. Kudryavtsev, V., Sikor, M., Kalinin, S., Mokranjac, D., Seidel, C.A. & Lamb, D.C. Combining MFD and PIE for accurate single-pair Forster resonance energy transfer measurements. *Chemphyschem* **13**, 1060-1078 (2012).
5. Kapanidis, A.N., Lee, N.K., Laurence, T.A., Doose, S., Margeat, E. & Weiss, S. Fluorescence-aided molecule sorting: analysis of structure and interactions by alternating-laser excitation of single molecules. *Proc Natl Acad Sci U S A* **101**, 8936-8941 (2004).
6. ATTO-Tec. Förster-Radius of ATTO-Dye-Pairs for FRET https://www.atto-tec.com/index.php?id=65&L=1 (accessed Jul. 2016).
7. Tosatto, L., Horrocks, M.H., Dear, A.J., Knowles, T.P., Dalla Serra, M., Cremades, N., Dobson, C.M. & Klenerman, D. Single-molecule FRET studies on alpha-synuclein oligomerization of Parkinson's disease genetically related mutants. *Sci Rep* **5**, 16696 (2015).
8. Cremades, N., Cohen, S.I., Deas, E., Abramov, A.Y., Chen, A.Y., Orte, A., Sandal, M., Clarke, R.W., Dunne, P., Aprile, F.A., *et al.* Direct observation of the interconversion of normal and toxic forms of alpha-synuclein. *Cell* **149**, 1048-1059 (2012).
9. Iljina, M., Garcia, G.A., Horrocks, M.H., Tosatto, L., Choi, M.L., Ganzinger, K.A., Abramov, A.Y., Gandhi, S., Wood, N.W., Cremades, N., *et al.* Kinetic model of the aggregation of alpha-synuclein provides insights into prion-like spreading. *Proc Natl Acad Sci U S A* **113**, E1206-1215 (2016).
10. Orte, A., Birkett, N.R., Clarke, R.W., Devlin, G.L., Dobson, C.M. & Klenerman, D. Direct characterization of amyloidogenic oligomers by single-molecule fluorescence. *Proc Natl Acad Sci U S A* **105**, 14424-14429 (2008).
11. Narayan, P., Orte, A., Clarke, R.W., Bolognesi, B., Hook, S., Ganzinger, K.A., Meehan, S., Wilson, M.R., Dobson, C.M. & Klenerman, D. The extracellular chaperone clusterin sequesters oligomeric forms of the amyloid-beta(1-40) peptide. *Nature Structural & Molecular Biology* **19**, 79-83 (2012).
12. Chiou, A., Hagglof, P., Orte, A., Chen, A.Y., Dunne, P.D., Belorgey, D., Karlsson-Li, S., Lomas, D.A. & Klenerman, D. Probing neuroserpin polymerization and interaction with amyloid-beta peptides using single molecule fluorescence. *Biophys J* **97**, 2306-2315 (2009).
13. Kapanidis, A.N., Heilemann, M., Margeat, E., Kong, X., Nir, E. & Weiss, S. Alternating-Laser Excitation of Single Molecules. In Single Molecule Techniques. A Laboratory Manual, eds. Selvin, P.R. and Ha, T. (Cold Spring Harbor Laboratory Press, New York (2008).
14. Schindelin, J., Arganda-Carreras, I., Frise, E., Kaynig, V., Longair, M., Pietzsch, T., Preibisch, S., Rueden, C., Saalfeld, S., Schmid, B., *et al.* Fiji: an open-source platform for biological-image analysis. *Nat Methods* **9**, 676-682 (2012).
15. Kelly, S.M., Jess, T.J. & Price, N.C. How to study proteins by circular dichroism. *Biochim Biophys Acta* **1751**, 119-139 (2005).
16. Morel, B., Varela, L., Azuaga, A.I. & Conejero-Lara, F. Environmental conditions affect the kinetics of nucleation of amyloid fibrils and determine their morphology. *Biophys J* **99**, 3801-3810 (2010).
17. Ruzafa, D., Morel, B., Varela, L., Azuaga, A.I. & Conejero-Lara, F. Characterization of oligomers of heterogeneous size as precursors of amyloid fibril nucleation of an SH3 domain: an experimental kinetics study. *PLoS ONE* **7**, e49690 (2012).
18. Doran, T.M., Kamens, A.J., Byrnes, N.K. & Nilsson, B.L. Role of amino acid hydrophobicity, aromaticity, and molecular volume on IAPP(20-29) amyloid self-assembly. *Proteins-Structure Function and Bioinformatics* **80**, 1053-1065 (2012).

19. Castello, F., Casares, S., Ruedas-Rama, M.J. & Orte, A. The First Step of Amyloidogenic Aggregation. *Journal of Physical Chemistry B* **119**, 8260-8267 (2015).

20. Wilkins, D.K., Grimshaw, S.B., Receveur, V., Dobson, C.M., Jones, J.A. & Smith, L.J. Hydrodynamic radii of native and denatured proteins measured by pulse field gradient NMR techniques. *Biochemistry* **38**, 16424-16431 (1999).

21. Morel, B., Casares, S. & Conejero-Lara, F. A single mutation induces amyloid aggregation in the alpha-spectrin SH3 domain: analysis of the early stages of fibril formation. *J Mol Biol* **356**, 453-468 (2006).

22. Amin, S., Barnett, G.V., Pathak, J.A., Roberts, C.J. & Sarangapani, P.S. Protein aggregation, particle formation, characterization & rheology. *Curr Opin Colloid Interface Sci* **19**, 438-449 (2014).
